# Changes in UK Pre-Schooler’s Mental Health Symptoms over the first year of the Covid-19 pandemic: data from Co-SPYCE Study

**DOI:** 10.1192/j.eurpsy.2023.321

**Published:** 2023-07-19

**Authors:** H. Dodd, S. Skripkauskaite, A. Shum, P. Waite, P. Lawrence

**Affiliations:** 1 Exeter Medical School, University of Exeter, Exeter; 2 Department of Experimental Psychology, University of Oxford, Oxford; 3 Department of Psychology, University of Southampton, Southampton, United Kingdom

## Abstract

**Introduction:**

The COVID-19 pandemic caused significant disruption to the lives of children and their families. Pre-school children may have been particularly vulnerable to the effects of the pandemic, with the closure of childcare facilities, playgrounds, playcentres and parent and toddler groups limiting their opportunities for social interaction at a crucial stage of development. Additionally, for parents working from home, caring for pre-school aged children who require high levels of support and care, was likely challenging

**Objectives:**

We aimed to conduct an intensive longitudinal study to examine trajectories of pre-schoolers’ mental symptoms in the United Kingdom during the first year of the COVID-19 pandemic.

**Methods:**

UK‐based parents and carers (n = 1520) of pre-school‐aged children (2 to 4 years) completed monthly online surveys about their pre-schoolers’ mental health between April 2020 and March 2021. The survey examined changes in children’s emotional symptoms, conduct problems and hyperactivity/inattention.

**Results:**

Pre-schoolers’ emotional problems and hyperactivity/inattention symptoms declined from April through summer 2020 and then increased again during the autumn and winter 2020/2021 as lockdowns were re-introduced. Pre-schoolers who attended childcare showed greater decline in symptom severity than those who did not. Older children, compared to younger, showed greater lability of emotion symptom severity. Attending childcare predicted lower symptom severity across all three domains of conduct problems, emotional symptoms, and hyperactivity/inattention, while the opposite pattern was observed for children whose parent had a mental health problem.

**Conclusions:**

Our findings reinforce the importance of examining pre-schoolers’ mental health in the context of micro and macro-level factors. Interventions focusing on family factors such as parent mental health, as well as continued provision of childcare, may have most potential to mitigate the impact of COVID-19 on young children’s mental health.

**Disclosure of Interest:**

None Declared

